# Extracellular Vesicles Secreted in Response to Cytokine Exposure Increase Mitochondrial Oxygen Consumption in Recipient Cells

**DOI:** 10.3389/fncel.2019.00051

**Published:** 2019-02-14

**Authors:** Ashley E. Russell, Sujung Jun, Saumyendra Sarkar, Werner J. Geldenhuys, Sara E. Lewis, Stephanie L. Rellick, James W. Simpkins

**Affiliations:** ^1^Department of Neuroscience, West Virginia University School of Medicine, Morgantown, WV, United States; ^2^Department of Physiology and Pharmacology, West Virginia University School of Medicine, Morgantown, WV, United States; ^3^Center for Basic and Translational Stroke Research, Department of Pharmaceutical Sciences, Rockefeller Neuroscience Institute, West Virginia University School of Pharmacy, Morgantown, WV, United States

**Keywords:** neuroinflammation, microRNAs, mitochondria, extracellular vesicles, TNF-α

## Abstract

Extracellular vesicles (EVs) are small, membrane-bound nanoparticles released from most, if not all cells, and can carry functionally active cargo (proteins, nucleic acids) which can be taken up by neighboring cells and mediate physiologically relevant effects. In this capacity, EVs are being regarded as novel cell-to-cell communicators, which may play important roles in the progression of neurodegenerative diseases, like Alzheimer’s disease (AD). Aside from the canonical physical hallmarks of this disease [amyloid β (Aβ) plaques, neurofibrillary tangles, and widespread cell death], AD is characterized by chronic neuroinflammation and mitochondrial dysfunction. In the current study, we sought to better understand the role of tumor necrosis factor-alpha (TNF-α), known to be involved in inflammation, in mediating alterations in mitochondrial function and EV secretion. Using an immortalized hippocampal cell line, we observed significant reductions in several parameters of mitochondrial oxygen consumption after a 24-h exposure period to TNF-α. In addition, after TNF-α exposure we also observed significant upregulation of two microRNAs (miRNAs; miR-34a and miR-146a) associated with mitochondrial dysfunction in secreted EVs. Despite this, when naïve cells are exposed to EVs isolated from TNF-α treated cells, mitochondrial respiration, proton leak, and reactive oxygen species (ROS) production are all significantly increased. Collectively these data indicate that a potent proinflammatory cytokine, TNF-α, induces significant mitochondrial dysfunction in a neuronal cell type, in part *via* the secretion of EVs, which significantly alter mitochondrial activity in recipient cells.

## Introduction

Alzheimer’s disease (AD) is a chronic neurodegenerative disease characterized by amyloid β (Aβ) plaques, neurofibrillary tangles, neuroinflammation, and mitochondrial dysfunction (Wyss-Coray and Rogers, 2012). Extracellular Aβ plaques activate microglia, prompting secretion of immunomodulatory molecules, including tumor necrosis factor-alpha (TNF-α; Hanisch, 2002). Elevated levels of this cytokine have been detected in the plasma (Fillit et al., 1991; Zuliani et al., 2007), cerebrospinal fluid (CSF; Tarkowski et al., 2003), and brains (Sharma et al., 2012) of AD patients when compared to healthy, age matched controls (AMCs). Further, several studies have implicated TNF-α as a key player in the formation of Aβ plaques (Blasko et al., 2000; Liao et al., 2004; Yamamoto et al., 2007), suggesting that this cytokine may participate in a vicious cycle of AD progression. TNF-α activates the transcription factor NF-κB (Schütze et al., 1995), which induces the transcription of many genes, including microRNAs (miRNAs); these small, non-coding RNAs function as gene repressors by base-pairing with complementary mRNAs and inhibiting protein translation, or accelerating the de-adenylation of the poly-A tail (Krek et al., 2005; Huntzinger and Izaurralde, 2011). Changes in expression of various miRNAs have been associated with inflammation and may influence AD progression.

Previous work from our laboratory assessed brain region-specific changes in miRNA expression, and observed significant upregulation of miR-34a and miR-146a in AD patients’ temporal cortices, when compared to AMCs (Sarkar et al., 2016). Promotor analysis revealed that various components of the NF-κB family bind to the promotor region of both miR-34a (Sarkar et al., 2016) and miR-146a (Taganov et al., 2006), suggesting that the activation of pro-inflammatory pathways could lead to increased transcription of these miRNAs. Interestingly, these miRNAs target several mRNAs, which encode for proteins of the mitochondrial electron transport chain (ETC; Dasgupta et al., 2015; Sarkar et al., 2016). Indeed, overexpression of miR-146a in primary microglial cells (Jun et al., unpublished observation) or miR-34a in primary neurons (Sarkar et al., 2016) significantly reduced mitochondrial function, and repressed expression of all of the associated mitochondrial proteins that were assessed (Sarkar et al., 2016). Inflammation-induced upregulation of NF-κB may lead to aberrant expression of miR-34a and -146a and lead to translational repression of ETC proteins, thus contributing to the widespread reductions in glucose metabolism and dysregulated mitochondrial function observed in AD brains.

Intercellular communication allows for the transfer of information from one cell to another. The secretion of extracellular vesicles (EVs) affords the opportunity for cells to send important signaling molecules out into the extracellular environment to convey vital messages to neighboring cells (Harding et al., 1983; Pan et al., 1985; Valadi et al., 2007; Tkach and Théry, 2016). As EVs are encapsulated by a lipid bilayer, their contents (including proteins, DNA, and RNA) are protected from degrading enzymes, allowing for delivery of functional cargo. During periods of neuroinflammation, concentrations of circulating EVs have been shown to be significantly upregulated, and within the context of AD, EVs have been shown to confer noxious molecules, including Aβ (Rajendran et al., 2006; Sardar Sinha et al., 2018), to recipient cells and thereby participate in the spreading of AD pathology and neuroinflammation.

Inasmuch as neuroinflammation clearly occurs in affected individuals, and likely occurs years prior to onset of clinical symptoms, it is difficult to model *in vitro*, the effects of long-term inflammation. In the current study, we sought to characterize the role of acute TNF-α exposure on phenotypes seen in AD, including mitochondrial dysfunction, increased miRNA expression (e.g., miR-34a and miR-146a; Sarkar et al., 2016), and altered EV secretion in an immortalized hippocampal cell line (HT-22). Here we demonstrate that direct exposure to TNF-α for 24-h significantly reduces mitochondrial oxygen consumption, induces cellular death, and induces differential expression of miR-34a and miR-146a both within cells, and in secreted EVs. We also assessed the functional outcomes of exposing TNF-α naïve cells to TNF-α-induced EVs and found significant increases in mitochondrial oxygen consumption, proton leak, and reactive oxygen species (ROS) production.

## Materials and Methods

### Cell Culture

The HT-22 immortalized mouse hippocampal cells were obtained from the Salk Institute for Biological Research (La Jolla, CA, USA). HT-22 cells were cultured in Hyclone Dulbecco’s modified Eagle’s medium (DMEM)/high glucose (Fisher Scientific, Waltham, MA, USA) with 10% fetal bovine serum (FBS; Atlanta Biologicals, Flowery Branch, GA, USA) and 1% penicillin/streptomycin (Fisher Scientific). When cells were at least 80% confluent, they were trypsinized and spun down at 325.5× *g* for 3 min. Cells were counted with a Nexcelom Bioscience Cellometer AutoT4 (Lawrence, MA, USA). Cell passages 5–18 were used for all experiments.

### Cytokine Reconstitution and Exposure

Recombinant mouse TNF-α was purchased from R&D Systems (Minneapolis, MN, USA) and reconstituted at 100 μg/ml in phosphate-buffered saline (PBS) containing 0.1% bovine serum albumin. Dilutions were made in Hyclone DMEM/high glucose with 10% exosome-depleted FBS (Fisher Scientific), and 1% penicillin/streptomycin to obtain concentrations of 0.1, 1, and 10 ng/ml. The 24-h time point for TNF-α exposure was chosen as preliminary data suggested that shorter exposure period did not result in mitochondrial dysfunction (data not shown). Longer exposure periods were not tested due to the potential complication of TNF-α-induced neurotoxicity on cell number, which could affect readouts of all of the assessed parameters in this study.

### EV Isolation From Cell Culture Media

Conditioned media was collected after a 24-h exposure to TNF-α and filtered through a Millex-AA Syringe Filter Unit, 0.80 μm (Millipore Sigma, Burlington, MA, USA) to remove cellular debris. EV isolation was performed as per the manufacturer’s instructions using either the ExoRNeasy Serum Plasma Maxi Kit (Qiagen, Germantown, MD, USA) utilized for RNA purification from EVs, or the ExoEasy Maxi Kit (Qiagen) for all other EV applications.

Briefly, 1 volume of filtered media was mixed with 1 volume of buffer XBP and then placed on a spin column and centrifuged at 500× *g* for 1 min. The flow-through was discarded and the column was washed with 10 ml buffer XWP and centrifuged at >3,000× *g* for 5 min. The column was then transferred to a new collection tube and the EVs were eluted with 700 μl QIAzol for downstream RNA purification, or 400 μl buffer XE for all other EV applications.

### Particle Size Distributions and Concentrations

To determine if changes in EV concentrations after exposure to TNF-α could account for alterations in mitochondrial function, EVs were isolated from HT-22 cell conditioned media using the ExoEasy Maxi Kit (as described above) and profiled with the NanoSight NS300 (Malvern, Westborough MA, USA). In the final isolation step using the ExoEasy Maxi Kit, EVs were eluted from the spin column membrane in 400 μl XE Buffer. Suspended EVs were diluted 1:200 in sterile filtered PBS for injection into the NanoSignt NS300 unit. Capture and analysis settings were manually set according to the manufacturer’s instructions. Particles were visualized using laser light scattering to quantify nanoparticles (10–1,000 nm) moving under Brownian motion as they pass through the flow chamber. The Nanoparticle Tracking Analysis (NTA) software generates particle size distributions and concentrations based on an analysis of both Brownian motion and light scattering observed from tracked particles.

### EV Marker Dot Blot

The presence of several EV markers was assessed to ensure that isolated particles from control or TNF-α exposure groups were indeed EVs. Protein concentration was measured using a microBCA kit (Fisher). BSA standards were prepared in the same solution EVs were eluted in (XE Buffer). EVs were diluted in 2% sodium dodecyl sulfide (SDS) at a ratio of 1:10 to a final volume of 150 μl, and transferred to a 96 well clear bottom assay plate. The standard curve was prepared using 150 μl of each, in duplicate. Working reagent was prepared in a 25:24:1 ratio of reagents A, B, and C, respectfully. One-hundred and fifty microliter working reagent was added to each of the standards and diluted EV samples, and incubated at room temperature for 2 h on a shaker. The plate was read using a BioTek Synergy H1 Hybrid reader at 562 nm absorbance.

The Exo-Check Exosome Antibody Array (System Biosciences) was used to detect the presence of several EV markers as per the manufacturer’s instructions. Briefly, from either control or TNF-α induced EVs, 200 μg protein was incubated with 600 μl Exosome Lysis buffer and vortexed. Next, the lysate mixture was combined with 9.4 mL Exosome Array Binding buffer and was then added to a pre-wet antibody array membrane and incubated overnight at 4°C on a shaker. The following day the membrane was washed and 10 mL Detection buffer was added to the membrane and incubated for 2 h on a shaker. Next, the membrane was washed 3× and 2 mL SuperSignal West Femto Chemi-luminescent Substrate (Fisher) developer solution was added and the membrane was imaged.

### Mitochondrial Functional Assessment

HT-22 cells were seeded at 5 k per well in an XF^e^96 cell culture microplate (Agilent, Santa Clara, CA, USA) for 24 h. Media was gently aspirated from the cells and replaced with 100 μl Hyclone DMEM/high glucose, supplemented with 10% exosome-depleted FBS and 1% penicillin/streptomycin, the various concentrations of TNF-α, or isolated TNF-α-induced EVs. For EV exposure, EVs were isolated from the media of HT-22 cells exposed to varying concentrations of TNF-α (0, 0.1, 1, and 10 ng/ml) for 24 h. As described above, EVs were isolated from 20 ml media of cytokine-exposed HT-22 cells and eluted from spin columns in 400 μl buffer XE. Assuming a high yield of recovery from a starting volume of 20 ml conditioned cell culture media, EVs eluted in 400 μl buffer XE would be concentrated 50×. To mimic *in vitro* EV concentrations from each cytokine exposure condition, EVs were diluted 50× in DMEM/high glucose, supplemented with 10% exosome-depleted FBS and 1% penicillin/streptomycin (i.e., 2 μl EVs suspended in buffer XE were diluted into 98 μl media per well). Various parameters of mitochondrial function were assessed using a Mito Stress kit, with the XF^e^96 Analyzer. Using Wave 2.4.0 Desktop software, all mitochondrial assessments were calculated with the Agilent Seahorse XF Cell Mito Stress Test Report Generator. Non-mitochondrial oxygen consumption rate (OCR) was calculated as the minimum rate measurement after rotenone/antimycin A injection. Basal respiration was calculated by subtracting non-mitochondrial OCR from the last rate measurement before oligomycin injection. Maximal respiration was calculated by subtracting non-mitochondrial OCR from the maximum rate measurement after trifluorocarbonylcyanide phenylhydrazone (FCCP) injection. Proton leak was calculated by subtracting non-mitochondrial OCR from the minimum rate measurement after Oligomycin injection. ATP production was calculated by subtracting the minimum rate measurement after Oligomycin injection from the last rate measurement before Oligomycin injection. Lastly, spare respiratory capacity was calculating by subtracting basal respiration from maximal respiration. All measurements are displayed percent changes from control. All wells with a basal respiratory rate below 40 pmol/min were excluded from analysis.

While glycolysis was not directly measured, data on the extracellular acidification rate (ECAR) is an additional measurement collected while completing the Mito Stress test. From these measurements, basal levels of glycolytic activity can be determined. To determine basal glycolysis, the third measurement, prior to the injection of oligomycin, was used. For cells treated with TNF-α, the average ECAR reading was calculated, and was used to calculate percent of control for each of the TNF-α treatment groups (0, 0.1, 1, and 10 ng/ml). For cells treated with EVs derived from TNF-α exposed cells, the cells treated with the XE buffer served as the control, and percent of control was calculated in the same way in each of the treatment groups (0, 0.1, 1, and 10 ng/ml).

### Lactate Dehydrogenase Assay

Cell death after cytokine or EV exposure was assessed using the Pierce^TM^ lactate dehydrogenase (LDH) Cytotoxicity Assay (Fisher Scientific) kit. Reaction substrates were prepared as per the manufacturer’s instructions. LDH assay was performed with the media from the XF^e^96 cell culture microplate. Forty-five minutes prior to the end of the 24-h exposure period, 10 μl 10× lysis buffer was added to one control well, and the plate was placed back in the incubator. At the conclusion of the exposure period, 50 μl of media was carefully removed from each well and transferred to a new 96 well clear bottom assay plate. The well exposed to 10× lysis buffer was excluded from the mitochondrial functional assessment. Next, 50 μl of the LDH reaction mixture was added to each well, and the plate was incubated for 30 min at room temperature, protected from light. The reaction was stopped by adding 50 μl of LDH stop solution to each sample. The plate was read using the BioTek Synergy H1 Hybrid reader (BioTek, Winooski, VT, USA) at absorbance of 490 nm and 680 nm. Cell death was calculated as a percentage of total death, relative to maximal cell death induced by treatment with the 10× lysis buffer.

### Intracellular and EV miRNA Analysis

Cells were seeded at 250 k per 10 cm cell culture dish and left to reach ~80% confluency over the course of 48 h. Media was gently aspirated from cells, and replaced by 10 ml of Hyclone DMEM/high glucose, supplemented with 10% exosome-depleted FBS and 1% penicillin/streptomycin, or the various concentrations of TNF-α. After a 24-h exposure period the media was collected for EV isolation, and cell lysates were collected using a Cell Lifter (Corning, Corning, NY, USA), washed with PBS, and pelleted by centrifugation at 325.5× *g* for 3 min, followed by the addition of 700 μl QIAzol Lysis Reagent for RNA purification (Qiagen).

RNA was purified from EVs eluted in 700 μl QIAzol by using the exoRNeasy Serum Plasma Maxi Kit (Qiagen). For all EV samples, 3.5 μl miRNeasy Serum/Plasma Spike-In Control (*C. elegans* miR-39; at a concentration of 1.6 × 10^8^ copies/μl) was added to each sample, followed by the addition of 90 μl chloroform and a 3 min incubation. Next the samples were centrifuged for 15 min at 12,000× *g* at 4°C. The upper aqueous phase (300 μl) was transferred to a new collection tube and mixed with 600 μl 100% ethanol. The solution was then placed on a RNeasy MinElute spin column and centrifuged at 12,000× *g* for 15 s. The flow through was discarded and 700 μl buffer RWT was added to the spin column and centrifuged at 12,000× *g* for 15 s. Two washes of 500 μl buffer RPE were then performed, the first lasting 15 s and the second lasting 2 min. The RNeasy MinElute spin column was then placed in a new collection tube and spun at 12,000× *g* for 5 min to dry the column membrane. Lastly, the column was placed in another collection tube and 28 μl RNase-free water was added to the center of the membrane and incubated for 1 min, and then centrifuged at 12,000× *g* for 1 min ending with the purified RNA eluted in the collection tube. Cellular RNA was purified using the miRNeasy Mini Kit (Qiagen) as per the manufacturer’s instructions, similar to the purification process for EVs, as described above.

RNA concentrations for each sample were measured using Nano drop 2,000 spectrophotometer (Thermo Scientific). For cDNA synthesis, 0.75 μg total RNA containing miRNAs was reverse transcribed using the miScript II RT kit (Qiagen). A 10 μl total volume reaction mix was made using 2 μl 5× miScript HiSpec Buffer, 1 μl 10× miScript Nucleics Mix, 1 μl miScript Reverse Transcriptase Mix, and 6 μl Template RNA/nuclease-free water. Prior to use for quantitative real-time PCR (qRT-PCR), cDNA was diluted in nuclease-free water, at a ratio of 1:10.

Previous work from our laboratory indicates that of a panel of miRNAs assessed, only miR-34a and miR-146a showed disease-stage related increases in expression in both human AD and triple transgenic AD mouse (3xTgAD) brains. Therefore, expression of miR-34a and miR-146a were profiled for both EV and intracellular levels using target specific miScript primer assays and the miScript SYBR^®^ Green PCR kit (Qiagen; 5 μl SYBR^®^ Green; 1 μl Universal Primer; 1 μl Target Primer; 3 μl diluted cDNA). qRT-PCR reactions were performed in triplicate for each sample, using the CFX384 Touch^TM^ RT PCR Detection System (Bio-Rad, Hercules, CA, USA) for 45 cycles as follows: 15 s at 94°C, 30 s at 55°C, 30 s at 70°C. Negative control reactions were included as wells containing only master mix and nuclease-free water (no template cDNA). All miRNA specific primers were miScript Primer Assays (Qiagen); Hs_RNU6-2_11 (MS00033740), Ce_miR-39_1 (219610), Hs_miR-34a (MS00003318), and Hs_miR-146a (MS00003535). The expression levels of target genes in EVs were standardized against those of the spike-in control, *C. elegans* miR-39 gene, detected in identical cDNA samples, while the expression levels of target genes in cell lysates was standardized against RNU6. Quantification of PCR amplified miRNA specific cDNA was done by comparative cycle threshold CT method (2^−ΒΒCT^).

### EV Uptake *via* Naïve Cells

To determine if naïve HT-22 cells have the capacity to take up cytokine-induced EVs, cells were incubated with EVs labeled with Exo-Red Exosome RNA Fluorescent Label (System Biosciences, Palo Alto, CA, USA). EVs isolated from all TNF-α exposure groups were labeled as per the manufacturer’s protocol. Briefly, ~100 μg protein of isolated EVs were incubated with 50 μl Exo-Red for 10 min at 37°C. ExoQuick-TC reagent (100 μl) was then added to the labeled EV suspension and mixed by gentle inversion. This solution was incubated on ice for 30 min, followed by centrifugation at 14,000 rpm for 3 min. The supernatant was removed and the EV pellet was resuspended in 500 μl PBS. Next, 100 μl labeled EVs were added to naïve HT-22 cells in a 6-well dish (~80% confluent), and imaged with the Nikon Swept Field Confocal microscope (Nikon, Melville, NY, USA) approximately 10 min after addition to naïve cells.

### Mitochondrial Membrane Potential Assay

Mitochondrial membrane potential (ψm) was measured using the tetramethylrhodamine, ethyl ester (TMRE; Abcam, Cambridge, United Kingdom) reagent. Cells were grown in a 96-well clear bottom black plate and exposed to TNF-α induced vesicles as described for the Seahorse assay. At the end of the experiment, media was gently aspirated from the wells and replaced by 100 μl of 60 nM TMRE in cell culture media, and incubated at 37°C for 20 min, protected from light. Cells were gently washed three times with warmed HBSS and fluorescence was measured on a Synergy HT multimodal plate reader with excitation 549 nm and emission 575 nm.

### Mitochondrial Superoxide Assay

The levels of mitochondrial superoxide in cells were determined using the MitoSOX^TM^ reagent (Fisher). Cells were grown in a 96-well clear bottom black plate and exposed to TNF-α induced vesicles as described for the Seahorse assay. At the end of the experiment, media was gently aspirated from the wells and replaced by 100 μl of 5 μm MitoSox in cell culture media and incubated for 10 min at 37°C, protected from light. Cells were gently washed three times with warmed HBSS and fluorescence was measured on a Synergy HT multimodal plate reader with excitation 510 nm and emission 580 nm.

### Hydrogen Peroxide Assay

The hydrogen peroxide (H_2_O_2_) assay was performed using the Amplex Red Kit (Invitrogen). Cells were grown in a 96-well clear bottom black plate, and exposed to TNF-α induced vesicles as described for the Seahorse assay. At the end of the experiment, 50 μL of the Amplex Red reagent was added to the media in the plate without washing, and incubated for 30 min at room temperature. The fluorescence of the Amplex Red reagent was measured on a Synergy HT multimodal plate reader with excitation 530 nm and emission 590 nm.

### Statistical Analyses

All biological experiments were repeated at least three times with *n* = 3–16 plates/wells per treatment. Results from the experiments are reported as means ± SEM. All quantitative data were assessed for significance using a one-way ANOVA with Dunnett’s *post hoc* test. All results were analyzed by GraphPad Prism 8.0 software (GraphPad Software, La Jolla, CA, USA). A *p* value < 0.05 was used to establish significance; significance presented as *p < 0.05, **p < 0.01, ***p < 0.001, and ****p < 0.0001.

## Results

### TNF-α Induces Mitochondrial Dysfunction and Cellular Death

**Figure 1:**
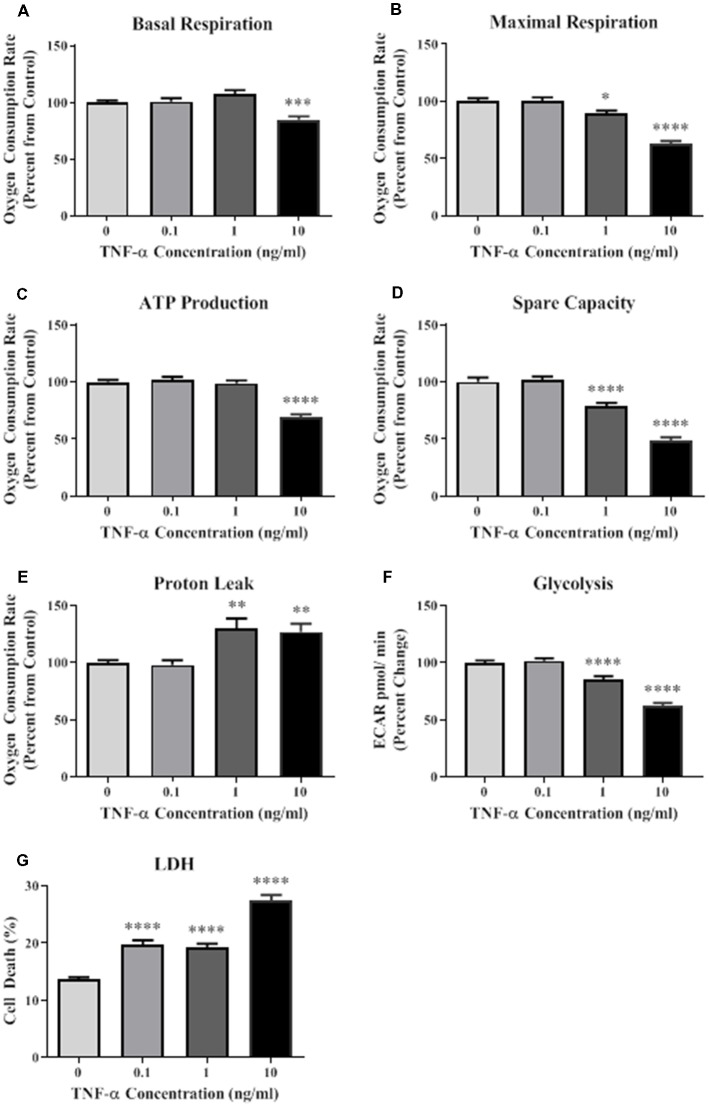
Tumor necrosis factor-alpha (TNF-α) exposure significantly reduces cellular bioenergetic function and increases cell death. After 24 h exposure to TNF-α, basal respiration (**A**; *F*_(3,292)_ = 11.90, *p* < 0.001), maximal respiration (**B**; *F*_(3,292)_ = 42.31, *p* < 0.0001), ATP production (**C**; *F*_(3,292)_ = 39.80, *p* < 0.0001), and spare capacity (**D**; *F*_(3,292)_ = 50.10, *p* < 0.0001) were significantly reduced at the highest two concentrations. Proton leak (**E**; *F*_(3,202)_ = 10.62, *p* < 0.0001) was significantly increased. Glycolysis was also significantly reduced after TNF-α exposure (**F**; *F*_(3,164)_ = 59.42, *p* < 0.0001). Lactate dehydrogenase (LDH) data indicate that significant cell death occurred at all concentrations of TNF-α exposure (**G**; *F*_(3,223)_ = 65.31, *p* < 0.0001). *p < 0.05, **p < 0.01, ***p < 0.001, and ****p < 0.0001.

A Seahorse mitochondrial stress test was conducted to assess mitochondrial function in HT-22 cells exposed to TNF-α for 24 h. Results indicate that TNF-α exposure reduced basal and maximal respiration, ATP production, and spare capacity in a dose-dependent manner, with significant reductions occurring at the two highest concentrations (1 and 10 ng/ml) compared to unexposed control cells (Figures 1A–D). These same concentrations of TNF-α induced significant proton leak (Figure 1E), which can reduce the ψm, and uncouple oxidative phosphorylation (Terada, 1990; Jastroch et al., 2010). Significant, dose-dependent decreases in basal glycolysis were also detected as a result of TNF-α exposure (Figure 1F). Exposure to all concentrations of TNF-α lead to cell death as shown by significant increases in LDH present in the conditioned culture media (Figure 1G). In view of the apparent cell death induced by increasing doses of TNF-α (Figure 1G), the observed decline in oxidative phosphorylation (Figures 1A–D) and glycolysis (Figure 1F) may be caused by the loss of cells in the assay.

### TNF-α Exposure Induces EV Secretion, but Not in a Dose-Dependent Fashion

**Figure 2:**
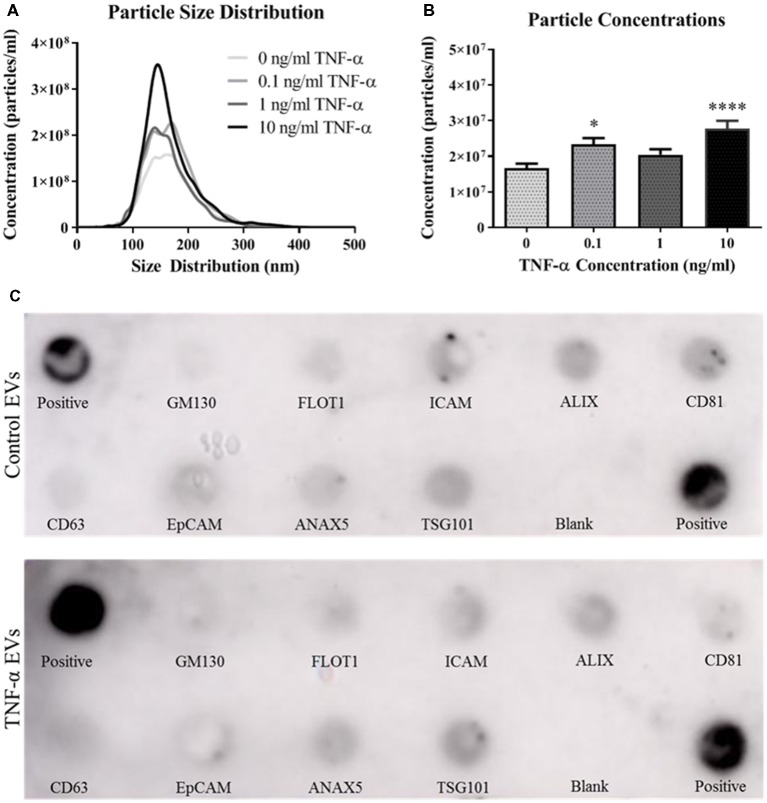
TNF-α exposure induces secretion of extracellular vesicles (EVs). Particle size distributions calculated from the NanoSight NS300 show expected size ranges for EVs (**A**; *F*_(3,3,996)_ = 7.06, *p* < 0.0001). Overall particle concentrations for each TNF-α exposure group indicate that TNF-α exposure induces vesicle secretion **(B)**. TNF-α induces EVs expressing canonical markers and are appropriately sized. Canonical EV proteins and their locations on a dot blot are shown **(C)**. Presence of proteins expressed for control vesicles (**C**, top panel) and TNF-α (10 ng/ml) induced vesicles (**C**, bottom panel) are shown. *p < 0.05, ****p < 0.0001.

We next assessed the ability of TNF-α exposure to induce EV secretion in this hippocampal cell line. Vesicles isolated from the conditioned media of TNF-α exposed cells were profiled using the NanoSight NS300. Interestingly, TNF-α exposure lead to increased EV particle number, however this effect does not appear to be dose-dependent (Figures 2A,B). Exposure to low (0.1 ng/ml) and high (10 ng/ml) concentrations of TNF-α significantly increased particle concentration, whereas exposure to 1 ng/ml TNF-α did not lead to particle concentrations above control. Additionally, TNF-α exposure did not alter vesicle size, as all EV size distributions were between ~80 and 300 nm in diameter, consistent with what has been shown in the literature (van Niel et al., 2018).

As recommended by the International Society for EVs (Lötvall et al., 2014), we probed for the presence of several canonical EV markers, including ALIX, CD81, CD63, and TSG101. Vesicles isolated from both the control and TNF-α exposure groups expressed all of the expected markers (Figure 2C) indicating that EVs were indeed being isolated in the vesicle preparation.

### TNF-α Alters miRNA Expression Intracellularly and in Secreted EVs

**Figure 3:**
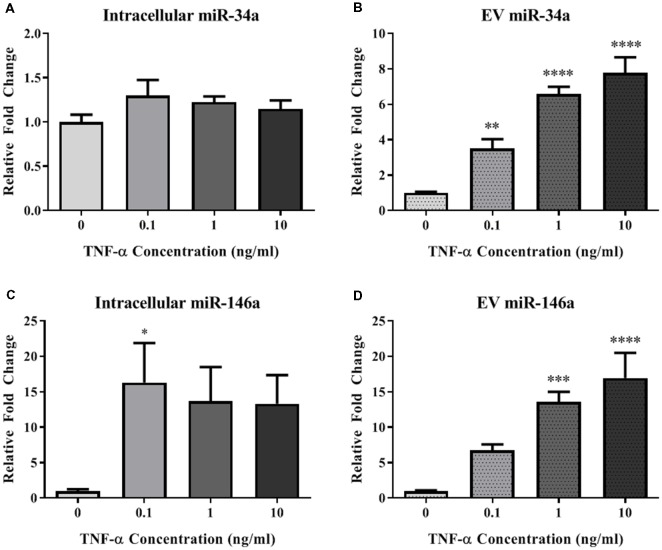
TNF-α significantly alters microRNA (miRNA) expression profiles. TNF-α exposure did not significantly alter intracellular expression of miR-34a (**A**; *F*_(3,32)_ = 1.29, *p* = 0.2951), but did induce a dose-dependent increase in miR-34a expression within EVs (**B**; *F*_(3,31)_ = 30.00, *p* < 0.0001). Conversely, TNF-α exposure induced intracellular upregulation of miR-146a which trended towards significance (**C**;* F*_(3,32)_ = 2.92, *p* = 0.0643), as well as a significant, dose-dependent increases within EVs (**D**; *F*_(3,31)_ = 13.15, *p* < 0.0001). *p < 0.05, **p < 0.01, ***p < 0.001, and ****p < 0.0001.

In order to determine the impact of acute TNF-α exposure on miRNA expression, we assayed miR-34a and miR-146a expression within cells and their secreted EVs 24-h after exposure. Interestingly, intracellular expression levels of miR-34a were not changed after exposure to TNF-α (Figure 3A), yet a dose-dependent significant upregulation of this miRNA was observed in the secreted EVs (Figure 3B). Intracellular miR-146a expression was significantly increased after TNF-α exposure with an apparent ceiling effect (Figure 3C), and was significantly increased in a dose-dependent fashion in secreted EVs (Figure 3D).

### EVs Are Taken Up by, and Enhance Mitochondrial Function in Recipient Cells

**Figure 4:**
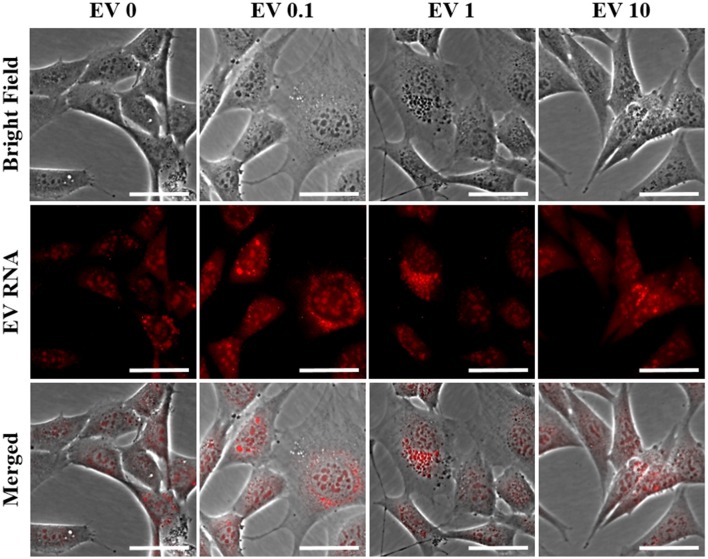
Naïve cell EV uptake and functional consequences. EVs derived from control and all concentrations of TNF-α (0, 0.1, 1, and 10 ng/ml) are taken up by naïve cells as evidenced by punctate staining of red-labeled EV RNA present within naïve cells. Scale bar = 40 μm.

**Figure 5:**
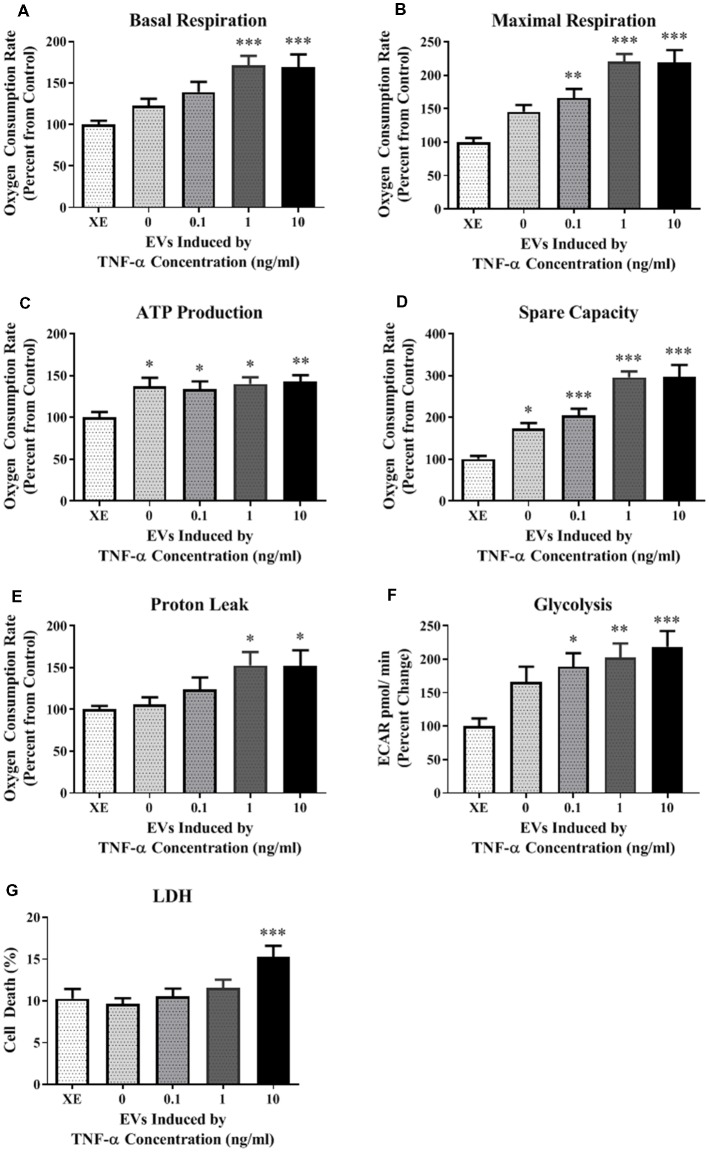
TNF-α induced EVs significantly alter energy metabolism. Data are depicted as significant differences related to XE buffer as the control. TNF-α induced EVs significantly increased basal respiration (**A**; *F*_(4,67)_ = 6.72, *p* = 0.0001) and maximal respiration (**B**; *F*_(4,67)_ = 15.05, *p* < 0.0001), while exposure to all vesicles significantly increased ATP production (**C**; *F*_(4,67)_ = 3.64, *p* = 0.0096) and spare capacity (**D**; *F*_(4,67)_ = 21.50, *p* < 0.0001). Proton leak was also significantly increased at the highest TNF-α concentrations (**E**; *F*_(4,67)_ = 3.07, *p* = 0.0221). Exposure to these vesicles significantly increased glycolysis as well (**F**; *F*_(4,84)_ = 5.024, *p* < 0.0011). Significant cell death only occurred at the highest concentration (**G**; *F*_(4,44)_ = 4.717, *p* = 0.003). Additional analyses using 0 ng/ml TNF-α exposed EVs as control were also conducted. When 0 EVs were used as the control, the effects of TNF-α-induced EVs remained significant for basal respiration (*F*_(3,55)_ = 3.614, *p* = 0.0187), maximal respiration (*F*_(3,55)_ = 7.607, *p* = 0.0002), spare capacity (*F*_(3,55)_ = 11.09, *p* < 0.0001), and LDH (*F*_(3,35)_ = 6.174, *p* = 0.0018). There were no significant responses to TNF-α-induced EVs for ATP production (*F*_(3,55)_ = 0.1857, *p* = 0.9057), proton leak (*F*_(3,55)_ = 2.266, *p* = 0.0909), or glycolysis (*F*_(3, 68)_ = 1.039, *p* = 0.3809). *p < 0.05, **p < 0.01, and ***p < 0.001.

Because the secreted EVs contained TNF-α dose-dependent increased expression of both miR-34a and miR-146a, we were interested in determining whether EV cargo could be delivered to TNF-α naïve recipient cells, and how this may affect their mitochondrial function. We exposed TNF-α naïve cells to EVs isolated from the media of TNF-α exposed cells whose RNA was fluorescently labeled; EVs delivered this RNA to naïve recipient cells (Figure 4). We then assessed mitochondrial function, using the XF^e^96 Mito Stress kit, in EV-recipient cells at 24 h after exposure. To our surprise, we observed significant, dose-dependent increases in mitochondrial oxygen consumption after EV exposure (Figures 5A–D). Interestingly, we also observed significant increases in proton leak (Figure 5E) suggesting that although oxidative phosphorylation is increased, mitochondria are likely not functioning optimally, as protons are leaking across the membrane. We also observed a significant dose-dependent upregulation of basal glycolysis after exposure to EVs (Figure 5F). Unlike direct exposure to TNF-α, only EVs induced from the highest concentration of TNF-α (10 ng/ml) caused significant cell death in naïve cells (Figure 5G). The enhanced oxidative phosphorylation (Figures 5A–D) and glycolysis (Figure 5F) seen after exposure to TNF-α-induced EVs is likely an underestimate of the bioenergetic function of the cells exposed to 10 ng/ml TNF-α-induced EVs, as this group experienced significant cell death (Figure 5G).

### EVs Increase ROS Production in Recipient Cells

**Figure 6:**
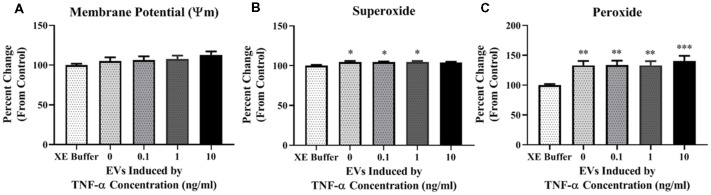
TNF-α induced EVs alter reactive oxygen species (ROS) production in recipient cells. Data are depicted as significant differences related to XE buffer as the control. EVs do not have a significant impact on the inner mitochondrial membrane potential (ψm; **A**; *F*_(4,115)_ = 1.287, *p* = 0.2794). Exposure of naïve cells to EVs significantly increases ROS production in the form of mitochondrial superoxide (**B**; *F*_(4,113)_ = 2.787, *p* = 0.0298), and hydrogen peroxide (H_2_O_2_; **C**; *F*_(4,85)_ = 5.329, *p* = 0.0007). Additional analyses using 0 ng/ml TNF-α exposed EVs as control were also conducted. There were no significant responses to TNF-α-induced EVs for membrane potential (*F*_(3,92)_ = 0.5786, *p* = 0.6305), mitochondrial superoxide (*F*_(3,90)_ = 0.07873, *p* = 0.9714), or H_2_O_2_ (*F*_(3,68)_ = 0.2021, *p* = 0.8946). *p < 0.5, **p < 0.01, and ***p < 0.001.

Due to the increased proton leak observed in the Seahorse assay following EV exposure, we next assessed mitochondrial ψm. We did not observe any significant changes in potential (Figure 6A) after a 24-h exposure period to TNF-α induced EVs.

We next assessed ROS production in cells exposed to TNF-α induced EVs. We observed a statistically significant increase in mitochondrial superoxide production when cells were incubated with EVs induced by 0, 0.1, and 1 ng/ml TNF-α, but not 10 ng/ml (Figure 6B); however, this result may be attributed to the significant amount of cell death observed in this group (Figure 5G). Additionally, we observed significant increases in H_2_O_2_ after exposure to EVs, with the highest amount generated in the 10 ng/ml TNF-α induced EV group (Figure 6C). As H_2_O_2_ has the capacity to induce neuronal cell death (Ricart and Fiszman, 2001), this may explain why we observed significant cellular death in this group (Figure 5G).

## Discussion

AD is characterized by neuroinflammation (Wyss-Coray and Rogers, 2012) and mitochondrial dysfunction (Swerdlow, 2018). In the present study, we observed that physiologically relevant levels of the proinflammatory cytokine TNF-α are capable of causing significant cellular death and mitochondrial dysfunction in hippocampal neurons after a 24-h exposure period. We also demonstrate that TNF-α induced EVs are taken up by naïve cells, and significantly alter mitochondrial activity in recipient cells. Specifically, we observed increases in mitochondrial respiration, glycolysis, proton leak, and ROS production in recipient cells after 24 h of EV exposure. Together, these data suggest that direct exposure of TNF-α to a neuronal cell type is highly toxic, and the vesicles secreted in response to this exposure are capable of inducing a stress response in neighboring cells.

We observed significant downregulation of mitochondrial oxidative phosphorylation and glycolysis in HT-22 cells exposed to TNF-α for 24 h. Under some conditions, when oxidative phosphorylation is inhibited or reduced, neurons can increase glycolysis as a protective, compensatory mechanism (Bas-Orth et al., 2017). However, with the decline in oxidative phosphorylation observed in this study, there is no compensatory increase in glycolysis. We additionally assessed expression of several ETC proteins involved in oxidative phosphorylation (including NDUFC2 of complex I, SHDC of complex II, UQCRB of complex III, and Cytochrome C oxidase of complex IV) after TNF-α exposure, and observed no significant changes in expression (data not shown). High concentrations of LDH in the media, along with reductions in bioenergetic measurements indicate that these observations are likely a result of cell death due to exposure to the cytotoxic protein, TNF-α.

We have previously shown that overexpression of miR-34a significantly reduces mitochondrial function in neuronal cells (Sarkar et al., 2016). In view of our evidence that miR-146a, but not miR-34a, was increased intracellularly in HT-22 cells, miR-146a may be responsible for the TNF-α induced mitochondrial impairment observed in these cells. Indeed, overexpression of miR-146a significantly reduces cellular bioenergetics (Jun et al., unpublished observation). Additionally, TNF-α exposure caused a dose-dependent increase in both miR-34a and miR-146a in secreted EVs. In line with other reports, these results suggest differential packaging and secretion mechanisms which are dependent on specific miRNAs (Abels and Breakefield, 2016). The results from the current study support the concept that some miRNAs (e.g., miR-34a) may be preferentially sorted out of cells (Jovičic and Gitler, 2017; Wei et al., 2017), however this interpretation is strictly speculative and requires further investigation.

As TNF-α exposure significantly elevated miR-34a and miR-146a expression within secreted EVs, we hypothesized that exposing naïve cells to TNF-α induced EVs would result in significant reduction of mitochondrial respiration due to miRNA-mediated repression of ETC protein translation. Interestingly, we observed significant increases in mitochondrial oxygen consumption in naïve cells exposed to these vesicles. In this study, only relative changes in miRNA expression were assessed, not definitive quantities. It has been postulated that there is fewer than one copy of an individual miRNA per EV (Chevillet et al., 2014; Wei et al., 2017), so it is likely that the absolute concentrations of miR-34a and miR-146a were not high enough to induce physiologically relevant repression of ETC proteins in recipient cells and cause reductions in oxygen consumption.

Several groups have also shown that exposure to EVs significantly increases mitochondrial respiration in recipient cells (Phinney et al., 2015; Sansone et al., 2017; Bland et al., 2018), and others have described that in response to stimulation, neurons are able to increase their rate of glycolysis (Yellen, 2018). This unexpected increase in mitochondrial activity may actually be a signal of mitochondrial stress, as we concomitantly observed dose-dependent increases in proton leak, suggesting that although oxidative phosphorylation is increased, the mitochondria are not actually functioning optimally. Indeed, a disproportionately large rate of mitochondrial oxygen consumption likely indicates an attempt to maintain ψm as proton leak across occurs (Jastroch et al., 2010). Spare respiratory capacity is an indicator of mitochondria’s ability to respond to stress (Hill et al., 2009). Here we observe significant elevation of spare capacity in cells exposed to EVs, which is likely the mechanism by which mitochondria were able to maintain ψm, despite undergoing significant proton leak. As mitochondria are the main generators of ROS, it is likely that the increased oxygen consumption incurred by maintaining ψm is responsible for significant ROS generation. Superoxide anions are quickly converted to less reactive H_2_O_2_ by superoxide dismutases (Lennicke et al., 2015), which may explain why we saw significantly more H_2_O_2_ than superoxide after EV exposure.

Our observation of altered mitochondrial function after a single exposure to TNF-α, or TNF-α induced EVs does not recapitulate the *in vivo* conditions of AD, as there is constant crosstalk between different cell types, and persistent, dynamic EV-cell interactions. However, the current findings suggest that neuronal, inflammation-derived EVs may acutely alter mitochondrial activity in recipient cells. The mitochondrial cascade hypothesis of AD posits that increased ROS induces mitochondrial DNA damage and reduces mitochondrial function; with time this initiates a “reset response” in which Aβ production is increased, leading to further generation of ROS in a vicious, positive feedback cycle (Swerdlow and Khan, 2004). While we have not identified the cargo within EVs that initiates this mitochondrial response, future studies will address this issue, as any component of EV-associated cargo may be responsible for these effects, e.g., direct transfer of specific proteins, mRNAs, or other miRNAs not assessed here.

Importantly, the current study provides novel insights on the role of inflammation-induced EVs in the inflamed and aging brain. The results underscore the importance of TNF-α, a canonical proinflammatory cytokine in the brain, as a regulator of mitochondrial function, miRNA expression, and EV secretion in neurons. Thus, TNF-α induced EVs may be drivers of the bioenergetic function and dysfunction throughout the brain in AD and other neuroinflammatory disorders.

## Data Availability

The datasets generated for this study are available on request to the corresponding author.

## Author Contributions

AR, SJ, SS, WG, and JS designed studies. AR and WG performed research. AR, SJ, SL, and SR analyzed data. AR and JS wrote the article.

## Conflict of Interest Statement

The authors declare that the research was conducted in the absence of any commercial or financial relationships that could be construed as a potential conflict of interest.

## Abbreviations

AD: Alzheimer’s disease
TNF-α: tumor necrosis factor-α
Aβ: amyloid β
AMCs: age matched controls
ETC: electron transport chain
EVs: extracellular vesicles
miRNA: microRNA
ROS: reactive oxygen species
ψm: mitochondrial membrane potential.
